# Improved Accessibility of Extracellular Vesicle Surface Molecules Upon Partial Removal of the Protein Corona by High Ionic Strength

**DOI:** 10.1002/jev2.70124

**Published:** 2025-07-14

**Authors:** András I. Försönits, Eszter Á. Tóth, Sára Jezsoviczky, Tünde Bárkai, Delaram Khamari, Alicia Galinsoga, Panna Királyhidi, Ágnes Kittel, Júlia Fazakas, Dorina Lenzinger, Hargita Hegyesi, Xabier Osteikoetxea, Tamás Visnovitz, Krisztina Pálóczi, Szilvia Bősze, Edit I. Buzás

**Affiliations:** ^1^ Department of Genetics, Cell‐ and Immunobiology Semmelweis University Budapest Hungary; ^2^ The Buda Hospital of the Hospitaller Order of Saint John of God Budapest Hungary; ^3^ Department of Rheumatology and Clinical Immunology Semmelweis University Budapest Hungary; ^4^ HCEMM‐SU Extracellular Vesicle Research Group Budapest Hungary; ^5^ HUN‐REN‐ELTE Research Group of Peptide Chemistry Budapest Hungary; ^6^ HUN‐REN‐SU Translational Extracellular Vesicle Research Group Budapest Hungary

**Keywords:** blood plasma, extracellular vesicle, flow cytometry, immunolabelling, protein corona

## Abstract

Recent studies have confirmed that a biomolecular corona forms around extracellular vesicles (EVs) in biofluids. However, there is limited data on how this adsorbed corona affects the accessibility of EV surface molecules. Here, we investigated various potential corona‐stripping conditions for their ability to affect the immune detection of EVs. First, we artificially formed an EV corona around nascent HEK293T‐PalmGFP cell‐derived large EVs (lEVs) by incubating them with Cy5‐labelled human plasma proteins. The co‐localisation rate of plasma proteins and lEVs decreased significantly upon high‐salt washing with NaCl, LiCl and KCl solutions, suggesting a considerable removal of the corona components. Additional evidence for corona modification was a significantly increased fluorescent annexin V binding to plasma lEVs and annexin V affinity capture of both THP1‐ and blood plasma‐derived lEVs upon high‐salt washing. A similar effect of high ionic strength was observed when THP1 lEVs were separated from a serum‐containing medium, which allowed for corona formation, but not when EVs were produced under serum‐free conditions. Using a MACSPlex kit and high‐salt washing for small EVs from plasma and THP1 conditioned medium, we also demonstrated significantly improved immunodetection of 15 and 9 out of 37 surface markers, respectively. In this Technical Note, we present evidence that modifying the protein corona around EVs can significantly affect the immune detection of specific EV markers.

## 1 Introduction

Extracellular vesicles (EVs) are lipid membrane‐enclosed extracellular particles released by cells ubiquitously in nature (Welsh et al. 2024; Buzas 2023). In the past decades, we have witnessed an explosive development of EV‐related research. EVs have been suggested to have significant roles in cell‐to‐cell communication and in cellular homeostasis. Moreover, an outstanding research interest is attracted to EVs because of their potential use as biomarkers in diagnostics as well as their application in therapy (Welsh et al. 2024).

Over the past decades, substantial efforts have been devoted to optimising EV separation from biological samples to better understand their molecular composition and functions (Welsh et al. 2024; Mathieu et al. 2019). A general observation of the EV research community is that EVs from biological fluids are often co‐separated with molecules considered contaminants of EV preparations (Welsh et al. 2024). Besides differential centrifugation, different other separation approaches have also been introduced to the EV field including size exclusion chromatography (SEC), density gradient ultracentrifugation (DGUC), tangential flow filtration, flow field flow fractionation, ion exchange chromatography and affinity capture approaches (Welsh et al. 2024). Although applying additional purification steps after an initial EV separation can result in increasingly pure EV preparations, it has become evident that no purification technique can yield pure EVs. In addition, there is a significant trade‐off between the yield and purity of EV preparations (Welsh et al. 2024).

On the other hand, in the field of artificial nanoparticles, it has been known since as early as 2007 that a protein corona is formed around nanoparticles in biofluids (Cedervall et al. 2007). Further studies confirmed that this adsorbed halo is, in fact, a complex biomolecular corona composed not only of proteins, but also of nucleic acids and lipids (Gunnarsson et al. 2018). Importantly, it has been shown that corona components could alter nanoparticle targeting, function and bioavailability (Xiao and Gao 2018).

However, it was only recently that the first direct experimental evidence confirmed a similar corona formation process in the case of extracellular vesicles (Tóth et al. 2021). The EV corona can be defined as a network of molecules associated with the EV surface rather than being part of the EV membrane or its internal cargo (Buzas 2022).

The significance of the EV corona is further supported by a recent semiquantitative model‐based estimation, which suggests a greater protein cargo on the surface of sEVs compared to their luminal protein cargo (Zendrini et al. 2022). We and others have shown earlier the association of DNA (Németh et al. 2017; Shelke et al. 2016; Lázaro‐Ibáñez et al. 2019) and low‐density lipoprotein (LDL) (Sódar et al. 2016; Busatto et al. 2020; Lozano‐Andrés et al. 2022) with the exofacial surface of EVs. More recently, we demonstrated a spontaneous adsorption of certain corona proteins on the surface of EVs in human blood plasma (Tóth et al. 2021). Numerous pieces of evidence have recently been published about the EV corona (Lozano‐Andrés et al. 2022; Wolf et al. 2022; Palviainen et al. 2020; Yerneni et al. 2022; Radeghieri et al. 2022; Liam‐Or et al. 2024) and that the proteins previously considered as co‐purified contaminants of EV preparations may now be recognised as components of the EV coronas (Welsh et al. 2024).

Despite our increasing knowledge about the functional significance and composition of EV coronas, many obvious questions remain unanswered. In the present study, we addressed the question of whether the modification of the protein corona may impact the affinity‐based detection of EV surface molecules. Here, we provide evidence that alteration of the corona by prior high‐salt washing of EVs, may significantly enhance the accessibility of certain EV surface molecules for detection by annexin V or antibodies.

## 2 Materials and Methods

### 2.1 Blood Collection

Altogether, 15 healthy subjects were enrolled in this study. Their mean age ±SD was 36.8 ± 5.7 years. The age range was 27–45 years, and the subjects included 8 males and 7 females (Table S1). Only individuals with no known history of chronic diseases were included in the analysis. Peripheral venous blood samples were collected with the ethics permission of the Hungarian Ministry of Human Capacities (EMMI) in agreement with the Hungarian Scientific and Research Ethics Committee (ETT TUKEB ‐ 25303‐5/2018/EÜIG). All included subjects signed an informed consent. Samples (18 mL) were collected by venipuncture of the cubital vein in acid‐citrate‐dextrose (ACD‐A) tubes (Grenier). The ACD‐A tubes were used to prevent in vitro vesiculation of blood cells in the collection tubes (György et al. 2014). In addition, blood was obtained from a patient with active rheumatoid arthritis (a 70 year old woman, Ethics Committee permission number 24101‐5/2020/EÜIG). The blood was processed within 1 h after collection. Until then, it was kept at room temperature (RT). In accordance with the recommendation of the International Society on Thrombosis and Haemostasis, the blood samples were centrifuged twice at 2500 × *g* at RT for 15 min to obtain platelet free plasma (PFP) (Lacroix et al. 2013). For quality control, plasma samples were tested for the absence of platelets using a Sysmex XE‐2100 automated haematology analyser.

### 2.2 EV Separation From PFP and Conditioned media of Cell Lines

For EV separation, 4–5 mL PFP was diluted two times with a 0.154 M NaCl solution (Fresenius Kabi) supplemented with 10 mM HEPES (Sigma–Aldrich) (NaCl‐HEPES). The diluted PFP was centrifuged at 18,300 × *g* at 12°C for 30 min (Hermle Z216MK, with a 220.87 rotor). The pellet was resuspended in NaCl‐HEPES, and centrifuged at 12,500 × *g* at 12°C for 20 min (Hermle Z216MK, with a 220.87 rotor, or an Eppendorf 5424 R centrifuge with an FA‐45‐24‐11 rotor). This pellet (considered to be enriched in large‐sized EVs (lEVs) with a diameter ∼200–800 nm (Welsh et al. 2024)) was resuspended in NaCl‐HEPES. After the first round of centrifugation, the supernatant was ultracentrifuged at 100,000 × *g* at 4°C for 70 min (Beckman Coulter Optima Max‐XP ultracentrifuge with MLA‐55 rotor). The pellet was washed once and resuspended in NaCl‐HEPES resulting in a preparation enriched in small EVs (sEVs) with a diameter of ∼80–200 nm.

For a bead‐based separation of EVs from PFP (4–5 mL), samples were diluted two times with NaCl‐HEPES, and were centrifuged at 18,300 × *g* for 30 min. The pellet was next resuspended in 300 µL of annexin‐binding buffer (AxBB, a NaCl‐HEPES buffer supplemented with 2.5 mM CaCl_2_). For each sample, 2 µL of Streptavidin MicroBeads along with 2 µL biotinylated annexin V (both from Miltenyi Biotec) was incubated for 30 min at RT while being rotated in a Fisherbrand Mini Tube Rotator (at 10 rpm). Four µL of the conjugated beads were then added to the 18,300 × *g* lEV pellet and the samples were incubated for 60 min at RT while rotated. The samples were then loaded onto magnetic separator μ Columns (MACS, Miltenyi Biotec). Prior to sample loading, the columns were pre‐treated with 100 µL 0.1% Triton‐X 100 and were washed 4 times with 150 µL AxBB. Next, the bead‐bound lEVs were captured in the column, were washed four times with 150 µL AxBB, were eluted with 100 µL of the same buffer and were kept refrigerated at 4°C until Nanoparticle Tracking Analysis (NTA) and flow cytometry analyses.

For the isolation and analysis of PFP‐derived sEVs, we used the MACSPlex Exosome Kit (Miltenyi Biotec). This multiplex bead‐based assay detects 37 surface proteins on sEVs in parallel. The protein content of the samples was determined using the bicinchoninic acid (BCA) protein assay (Micro BCA Protein Assay Kit, Thermo Fisher Scientific). The antibody‐coated fluorescent bead populations of the kit were incubated with 10 µg protein containing sEVs, followed by an overnight protocol of the manufacturer for 1.5 mL tubes.

For EV separation from HEK293T‐PalmGFP (generous gift of Charles Lai), cells were grown in high glucose DMEM (Sigma) medium, containing 1% Antibiotic Antimycotic Solution for Cell Culture (EMD Millipore) and 1% glutamine (Gibco) in T75 flasks in serum‐free medium for 24 h until confluency (7.8–8.2 × 10^6^/mL). Prior to EV separation, cell viability was assessed by annexin V‐FITC binding by flow cytometry. Next, 12 mL conditioned medium was used to separate lEVs. To this end, cells and larger particles were removed by 300 × *g* 10 min 25°C and 2000 × *g* 20 min at 16°C centrifugations, respectively. The supernatant was filtered through a 0.8 µm pore size filter (Millipore), and the flow‐through was centrifuged at 12,500 × *g* at 12°C for 20 min as described previously (Tóth et al. 2021).

For EV separation from the THP1cell line (ATCC), cells were cultured at 3.5–5 × 10^5^/mL concentration in RPMI 1640 (Sigma) containing 10% foetal bovine serum (FBS) (Gibco, BioSera), 1% Antibiotic Antimycotic Solution for Cell Culture (EMD Millipore) and 1% glutamine (Gibco). If separation was carried out from a serum free medium. The cells were transferred to FBS‐free medium 24 h before separation. Prior to EV separation, cell viability was assessed by annexin V‐FITC binding by flow cytometry.

For differential centrifugation‐based separation, we used the same method as described above for HEK293T‐PalmGFP‐derived EV separation; for measuring zeta potential, we also separated lEVs and sEV from 10% FBS containing conditioned medium of 8 × 10^6^/mL A549 and H1975 lung cancer cell lines (ATCC).

For affinity EV separation, we used annexin V‐covered magnetic microbeads prepared as described above. First, cells and larger particles were removed by 300 × *g* 10 min 25°C and 2000 × *g* 20 min at 16°C centrifugations, respectively. The supernatant was filtered through a 0.8 µm pore size filter (Millipore), and the flow‐through was centrifuged at 12,500 × *g* at 12°C for 20 min as described previously (Tóth et al. 2021). We resuspended the annexin V positive lEV‐enriched pellet in 100 µL NaCl‐HEPES.

### 2.3 High‐Salt Washing and Different Other Approaches to Alter the Protein Corona of EVs

In an attempt to modify the EV corona, after the first centrifugation step at 12,500 × *g* for cell culture‐derived and 18,300 × *g* for PFP‐derived lEVs, or 100,000 × *g* for sEVs, the pellets were resuspended in solutions containing (i) 0.1 M NaCl‐HEPES (as control), (ii) 1.5 M NaCl, (iii) 1.5 M LiCl, (iv) 1.5 M KCl, (v) 1.5 mg/mL EDTA, (vi) 25 mM β‐2‐mercaptoethanol, (vii) or 0.1% Tween 20, dissolved in NaCl‐HEPES (pH = 7). Following resuspension, the EVs were pelleted at 12,500 × *g* for 20 min for lEVs or 100,000 × *g* 70 min for sEVs. In the case of affinity separations, the sample incubated with the capture molecules were already washed with these corona‐altering solutions.

### 2.4 Preparation of Cy5‐labelled Human Plasma Proteins to Build an EV Corona In Vitro

PFP depleted in lEVs was prepared from 8 mL ACD‐A anticoagulated human blood plasma of a healthy young male adult (33 y) as described above, followed by an overnight centrifugation at 18,300 × *g* at 4°C.  Two hundred microgram protein‐containing lEV‐depleted PFP was labelled with a Cy5 Conjugation Kit (Fast)—Lightning‐Link (ABCAM) according to the manufacturer's instructions. Unbound Cy5 molecules were quenched by the Quencher reagent of the kit. Of note, the lEV‐depleted plasma used for the lEV corona formation still contained sEVs and lipoproteins. Thus, in our experiments, all the lEV‐depleted plasma proteins were labelled (irrespective of their soluble, vesicular or lipoprotein nature) given that they could all contribute to the EV corona formation.

### 2.5 Confocal Microscopy of the EV Corona

The green fluorescent HEK293TPalm‐GFP lEVs were incubated in 50 µL, 50 ng/µL Cy5 (Abcam)‐labelled PFP for 60 min at RT in the dark. After this incubation, the samples were washed with different potential protein corona‐stripping solutions and were resuspended in 50 µL 0.1 M NaCl‐HEPES. As an alternative approach, we subjected labelled plasma protein‐coated lEVs to SEC (Izon), according to the manufacturer's protocol. After incubating the HEK293‐PalmGFP‐ derived lEV pellet with the Cy5‐labelled lEV‐depleted PFP, we ran it on a 70 nm pore size gen2 qEVoriginal column. We collected 10 fractions (200 µL each) and measured both the GFP signal and the Triton‐sensitivity with flow cytometry (Cytoflex S, Beckman Coulter). Fractions 4–7 contained GFP positive, Triton sensitive events. The two fractions (5,6) with largest fluorescent intensity were pooled and placed on precoated coverslips. We pre‐coated coverslips with 100 µL 0.1 mg/mL poly‐D‐lysine (Sigma), pipetted the 50 µL lEV‐s onto the slides, and incubated them in dark in 24 well plates for 20 min at RT. After the incubation, we fixed the samples in 4% PFA and mounted the slides with ProLong Diamond Antifade Mountant (ThermoFisher). For imaging, we used a Leica TCS SP8 Confocal Laser Scanning microscope (Leica, Germany). To assess corona‐coated EVs, the co‐localisation rate of GFP positive vesicles and Cy5‐labelled blood plasma proteins per each green fluorescent particle was analysed using the Leica Application Suite X (LAS X) software in randomly selected four microscopic fields using a 63x objective lens. Statistical analysis was carried out by the GraphPad Prism 10 software (GraphPad Software Inc.).

### 2.6 Flow Cytometry Analysis of EV Surface Markers

lEVs separated from PFP samples by differential centrifugation or annexin V‐based affinity capture were analysed using a CytoFLEX S flow cytometer (Beckman Coulter, USA). We labelled lEVs in 250 µL of AxBB with annexin V‐FITC (from SONY). Dye‐only and unstained controls were used during the analysis. Furthermore, after the measurements, we applied 0.1% Triton‐X 100 lysis to prove the vesicular nature of the annexin V positive events (Osteikoetxea et al. 2015). In the case of serum free conditioned medium‐derived THP1 EVs, equal numbers of washed and control particles were analysed by flow cytometry. The particle count was determined by NTA (ZetaView, ParticleMetrix). The median fluorescence intensity of the samples was used in the statistical tests.

For sEVs, the flow cytometry measurements were carried out by using a MACSPlex Exosome Kit on a CytoFLEX S flow cytometer (Beckman Coulter, USA). The sEVs bound to antibody‐coated beads were stained with APC‐conjugated anti‐CD63, CD9 and CD81 antibodies. The median fluorescence intensities of the bead populations were used in our statistical analysis (Welsh et al. 2020). The normalisation basis of the fold change calculation was always the fluorescence intensity of the control half of a given sample pair.

### 2.7 Nanoparticle Tracking Analysis

To compare size distributions and zeta potentials of high‐salt washed and control lEVs and sEVs, we used a ZetaView Z‐NTA instrument (ParticleMetrix) with a ZetaView Analyze 8.05.10 software. Samples were measured within 2 h after separation. We used 0.1 µm filtered NaCl‐HEPES at 15,000 µS/cm conductivity for sample dilution, with a 520 nm laser. The measurement temperature was 25°C. The camera settings were as follows: auto expose, sensitivity (se): 80, shutter (sh): 100, gain: 28.8, offset: 0, while the analysis parameters were: max area: 10,000, min area: 5, min brightness: 20. Each sample was diluted to contain the optimal number (30–150) of particles/frame for the measurement. The samples were diluted in 0.1 µm filtered 0.1x PBS (pH 6.9) at 2000 µS/cm conductivity for the zeta potential measurements. The measurement temperature was 25°C. The camera settings were the following: sh: 70 / se: 85 / frame rate (fr): 30; continuous, 5 cycles, 2 positions, respectively. Each sample was diluted to contain the optimal number (150–200) of particles/frame for the measurements.

### 2.8 EV Uptake Assay

To assess the impact of high salt washing on the uptake of sEVs, we used the HEK293T‐PalmGFP cell line as a source of sEVs. The fluorescent sEVs were separated from the 10% FBS containing conditioned medium of 10^7^ cells with differential ultracentrifugation as described above. Next, they were washed in either 1.5 M NaCl or physiological NaCl‐HEPES solutions. The final pellets were resuspended in 60 µL NaCl‐HEPES. For EV uptake, we used THP1 cells cultured as described above. The EV uptake of 20,000 THP1 cells were measured after 0, 30 and 60 min after co‐incubation with 2 × 10^9^ HEK293T‐PalmGFP sEV particles (measured by NTA) with or without high salt washing. To assess the uptake, the percentage of GFP positive cells were determined using a CytoFLEX S flow cytometer (Beckman Coulter, USA). In an alternative approach, we collected the serum free conditioned medium of 10^7^ HEK293T‐PalmGFP cells and isolated sEVs with differential ultracentrifugation as described above. We incubated these nascent sEVs in 1 mL of EV‐depleted PFP sample obtained from an active rheumatoid arthritis patient (DAS score 5,9). For EV depletion, the supernatant of 5 mL lEV‐depleted PFP was further centrifuged at 100,000 × *g* for 16 h. We divided the human plasma‐coated sEVs into two aliquots, and applied high salt washing to one of the aliquots and normal salt washing to the other aliquot. We assessed the uptake by THP1 cells as described above.

### 2.9 Assessment of EV Cargo Delivery

To detect the transfer of Cas9 and sgRNA by either high salt‐washed or physiological salt‐washed sEVs, a fluorescent reporter construct and a sgRNA loading construct were generated. The fluorescent reporter construct was generated by PCR‐based cloning of a LoxP‐tdTomato‐STOP‐LoxP‐eGFP sequence from an earlier publication (Cat#17787, Addgene, Watertown, USA) (Muzumdar et al. 2007) into a mammalian expression vector. The sgRNA loading construct was designed with the following sequence, GCATTATACGAAGTTATATTA and cloned in a sgRNA expression plasmid under human U6 promoter. This sequence targets the LoxP sequences found in the fluorescent reporter leading to excision of tdTomato and expression of eGFP. These plasmids are available to other researchers upon request to the authors. To study EV transfer of Cas9 and sgRNA, a cell line was generated for stable expression of the fluorescent reporter. The reporter cell line was generated by transfection of HEK293T cells using PEI max with mammalian expression vector, followed by selection with 2 µg/mL Puromycin (Cat# A1113803, ThermoFisher). For the isolation of Cas9 and sgRNA loaded EVs, we followed a previously described method (Osteikoetxea et al. 2022). Briefly, prior to EV isolation, HEK293T cells were transfected with plasmids for Cas9 and sgRNA loading in a ratio of 4:1 using PEI max transfection reagent (Cat# 24765‐1, Polysciences, Warrington, USA). Cas9‐loaded sEVs were isolated from 10% FBS‐containing conditioned medium of 2 × 10^8^ transfected HEK293T cells by differential ultracentrifugation as described above and after the first round of 100,000 × *g* 70 min, the sEV pellet was divided into two parts; one half was washed with 1.5 M NaCl solution (pH7), while the other half was washed with physiological NaCl‐HEPES solution at 100,000 × *g* 70 min. The pellets were resuspended in 60 µL NaCl‐HEPES.

HEK293T cells expressing the fluorescent reporter construct were seeded at 30,000 cells/well density onto 96‐well plates (Thermo Scientific), and Cas9‐loaded sEVs were added to the cells (108‐1011 EVs/well, and the concentrations were determined by NTA). The percentage of GFP positive cells was determined using a CytoFLEX S flow cytometer (Beckman Coulter, USA) after 48 h.

### 2.10 Fluorimetry

To confirm that high salt washing indeed eliminated some components of the protein corona from the surface of EVs, we centrifuged the Cy5‐labelled plasma protein corona‐coated EVs at 12,500 × *g* and assessed the 12,500 × *g* supernatant in the 670 nm emission peak of Cy5 using an LS‐50B Spectrometer (PerkinElmer Instruments).

### 2.11 Transmission Electron Microscopy (TEM)

EV‐enriched pellets were fixed with 4% PFA in PBS for 60 min at RT and were analysed by TEM. After washing with PBS, the EV preparations were postfixed in 1% osmium tetroxide (OsO4, Taab, Aldermaston, Berks, UK). This was followed by rinsing with distilled water. The pellets were dehydrated in graded ethanol, including block staining with 1% uranyl‐acetate in 50% ethanol for 30 min, and were embedded in Taab 812 (Taab). An overnight polymerisation of samples at 60°C was followed by sectioning, and the ultrathin sections (50–70 nm) were analysed using a Hitachi 7100 electron microscope (Hitachi Ltd., Japan) equipped by Veleta, a 2000 × 2000 MegaPixel side‐mounted TEM CCD camera (Olympus) (Tóth et al. 2021).

sEVs separated from serum‐free conditioned medium, were detected by negative‐positive contrasting as described by Théry et al. (2006) with minor modifications. Briefly, drops of EV‐samples were placed on formvar‐coated grids and for contrasting we used UranyLESS (Electron Microscopy Sciences—EMS, USA, 22409) reagent mixed with methyl cellulose solution. Grids were analysed using a JEM1010 (JEOL) transmissional electronmicroscope equipped with a MSYS Mega View 3 digital camera.

### 2.12 Statistics and Graphic Software

We used the GraphPad Prism 10 software (GraphPad Software Inc.). Prior to analysis, the normality of the distribution of the data points was tested. Then, a pairwise analysis of the datasets was carried out with Student's paired *t*‐test or with Wilcoxon's matched‐pairs signed rank test, based on the data distribution with *p < 0.05, **p < 0.01, ***p < 0.001 significance levels. In the case of the uptake and cargo delivery assays, two‐way ANOVA test was used with Geisser‐Greenhouse correction. The schematic figures were created by Biorender (biorender.com).

## 3 Results

### 3.1 Removal of EV Corona Proteins by High Ionic Strength

**FIGURE 1:**
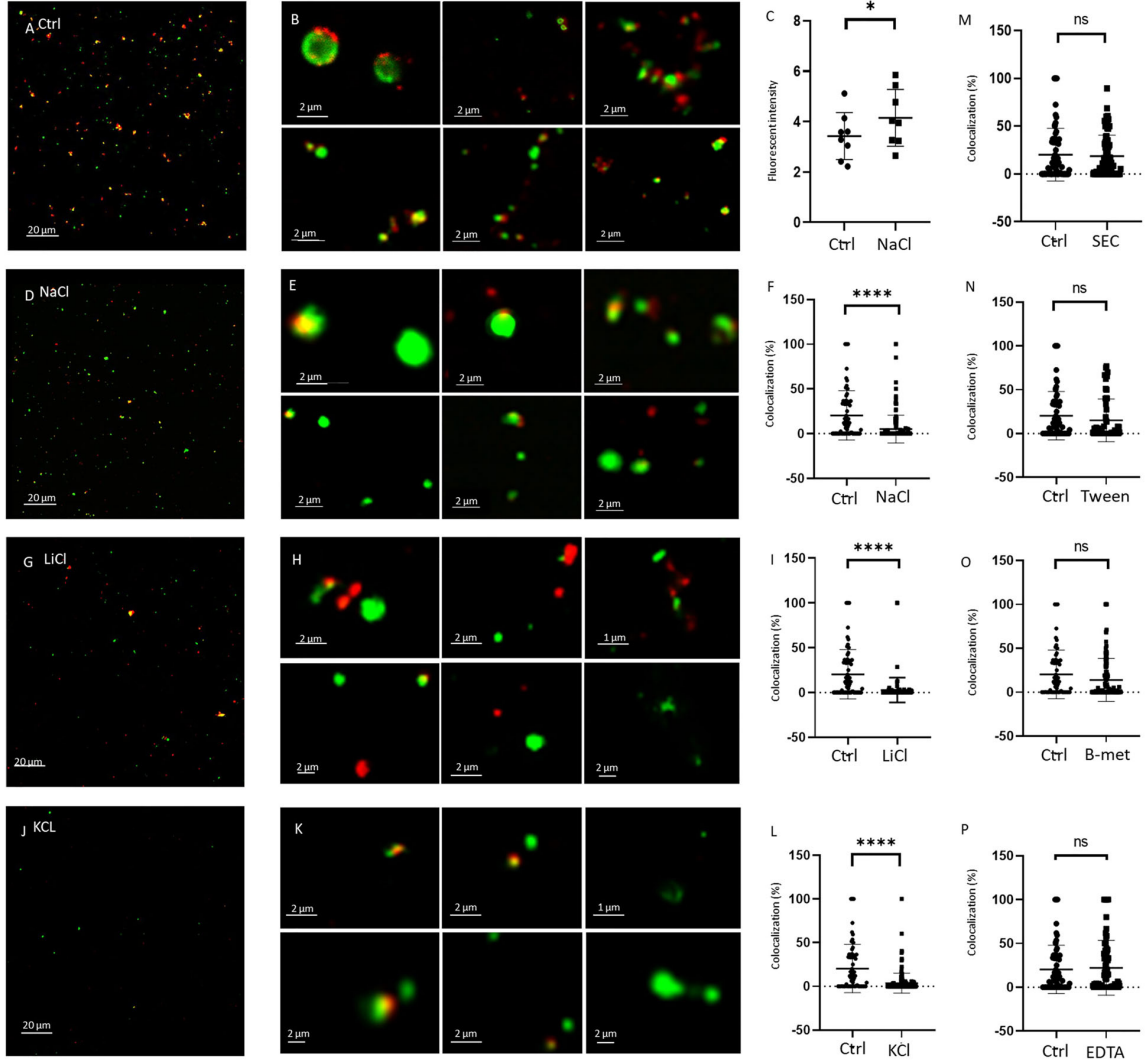
Fluorescently labelled blood plasma protein‐coated HEK293T‐PalmGFP lEVs. Effect of different washing solutions as compared to physiological salt washing. (A) Confocal microscopic images of plasma protein corona‐coated lEVs. lEVs were separated from serum‐free conditioned medium of HEK293T‐PalmGFP cells, were incubated with Cy5‐labelled plasma proteins and were washed with physiological salt solution. (B) Close‐up images of HEK293T‐PalmGFP lEVs coated with Cy5‐labelled plasma protein corona after washing with physiological salt solution. (C) HEK293T‐PalmGFP lEVs were incubated with Cy5‐labelled plasma proteins and were centrifuged at 12,500 × *g* with or without high salt washing. The fluorescence intensity of the supernatant was determined at 667 nm (*n* = 8, paired *t*‐test, *p* = 0.0146). (D) Wide‐field image of Cy5‐labelled plasma protein‐coated HEK293T‐PalmGFP lEV after high‐salt (1.5 M NaCl) washing. (E) Close‐up images of HEK293T‐PalmGFP lEVs coated with Cy5‐labelled plasma protein corona washed with high‐salt solution. (F) Co‐localisation of individual GFP positive lEVs and Cy5‐labelled plasma proteins with and without high‐salt washing. The co‐localisation percentages are shown in the y axis. (*n* = 172 lEVs, Mann–Whitney test, *p* < 0.0001). (G) Wide‐field image of Cy5‐labelled plasma protein‐coated HEK293T‐PalmGFP lEVs after high LiCl (1.5 M LiCl) washing. (H) Close‐up images of HEK293T‐PalmGFP lEVs coated with Cy5‐labelled plasma protein corona after washing with high lithium‐salt solution. (I) Co‐localisation of individual GFP positive lEVs and Cy5‐labelled plasma proteins with and without high LiCl washing. The co‐localisation percentages are shown in the y axis (*n* = 190 lEVs, Mann–Whitney test, *p* < 0.0001). (J) Wide‐field image of Cy5‐labelled plasma protein‐coated HEK293T‐PalmGFP lEV after high potassium‐salt (1.5 M KCl) washing. (K) Close‐up images of HEK293T‐PalmGFP lEVs coated with Cy5‐labelled plasma protein corona after washing with high KCl solution. (L) Co‐localisation of individual GFP positive lEVs with Cy5‐labelled plasma proteins with and without high KCl washing. The co‐localisation percentages are shown in the y axis (*n* = 170 lEVs, Mann–Whitney test, *p* < 0.0001). (M) Co‐localisation of individual GFP positive lEVs and Cy5 upon isolation by SEC and by differential centrifugation. The co‐localisation percentages are shown in the y axis. (*n* = 74 lEVs, Mann–Whitney test, *p* = 0.68). (N) Co‐localisation of individual GFP positive lEVs and Cy5‐labelled plasma proteins with and without washing with 0.1% Tween20. The co‐localisation percentages are shown in the y axis (*n* = 68 lEVs, Mann–Whitney test, *p* = 0.15). (O) Co‐localisation of individual GFP positive lEVs and Cy5‐labelled plasma proteins with and without washing with 25 µM β‐2‐mercaptoethanol. The co‐localisation percentages are shown in the y axis (*n* = 128 lEVs, Mann–Whitney test, *p* = 0.06). (P) Co‐localisation of individual GFP positive lEVs and Cy5‐labelled plasma proteins with and without washing with 15 mg/mL EDTA. The co‐localisation percentages are shown in the y axis (*n* = 88 lEVs, Mann–Whitney test, *p* = 0.97).

We hypothesised that the presence of a protein corona around the surface of EVs may interfere with the accessibility of exofacial EV molecules. To test this hypothesis, we separated and analysed EVs from blood plasma with and without high‐salt washing. We presumed that this washing step may modify the protein corona, and thus, may improve the accessibility of the EV surface. In order to prove this concept, we incubated lEVs released by the green fluorescent HEK293T‐PalmGFP cell line with Cy5‐labelled lEV‐depleted PFP proteins for 1 h. Figure 1A,B show the isolated corona‐coated lEVs. Next, we washed the coated lEV‐s with different solutions to modify the protein corona. As shown in Figure 1D,E,G,H,J,K, high salt washing (1.5 M concentration of NaCl, LiCl and KCl, respectively) reduced the amount of EV surface‐associated Cy5‐labelled plasma proteins as compared to physiological salt washing. As an indirect approach, we also measured the supernatant of the high‐salt‐washed and control (physiological salt solution‐washed) lEVs, and found that the high‐salt containing supernatant had a significantly increased fluorescent signal at 667 nm (paired *t*‐test, *p* < 0.01, Figure 1C). 

Based on image analysis of lEVs, we could detect a significant decrease in the co‐localisation of the corona‐forming Cy5‐labelled PFP proteins and the green fluorescent membrane of lEVs (Mann–Whitney test, *p* < 0.0001, Figure 1F,I,L).

We have also applied different other approaches to alter the protein corona by isolating EVs with SEC (Figure 1M), washing with Tween‐20 (Figure 1N), β‐2‐mercaptoethanol (Figure 1O) and EDTA (Figure 1P). Yet only washes with high salt concentrations had a significant effect.

### 3.2 Detection and Affinity Capture of THP1 Cell Line‐Derived lEVs With and Without the Alteration of the Protein Corona by High‐Salt Washing

**FIGURE 2:**
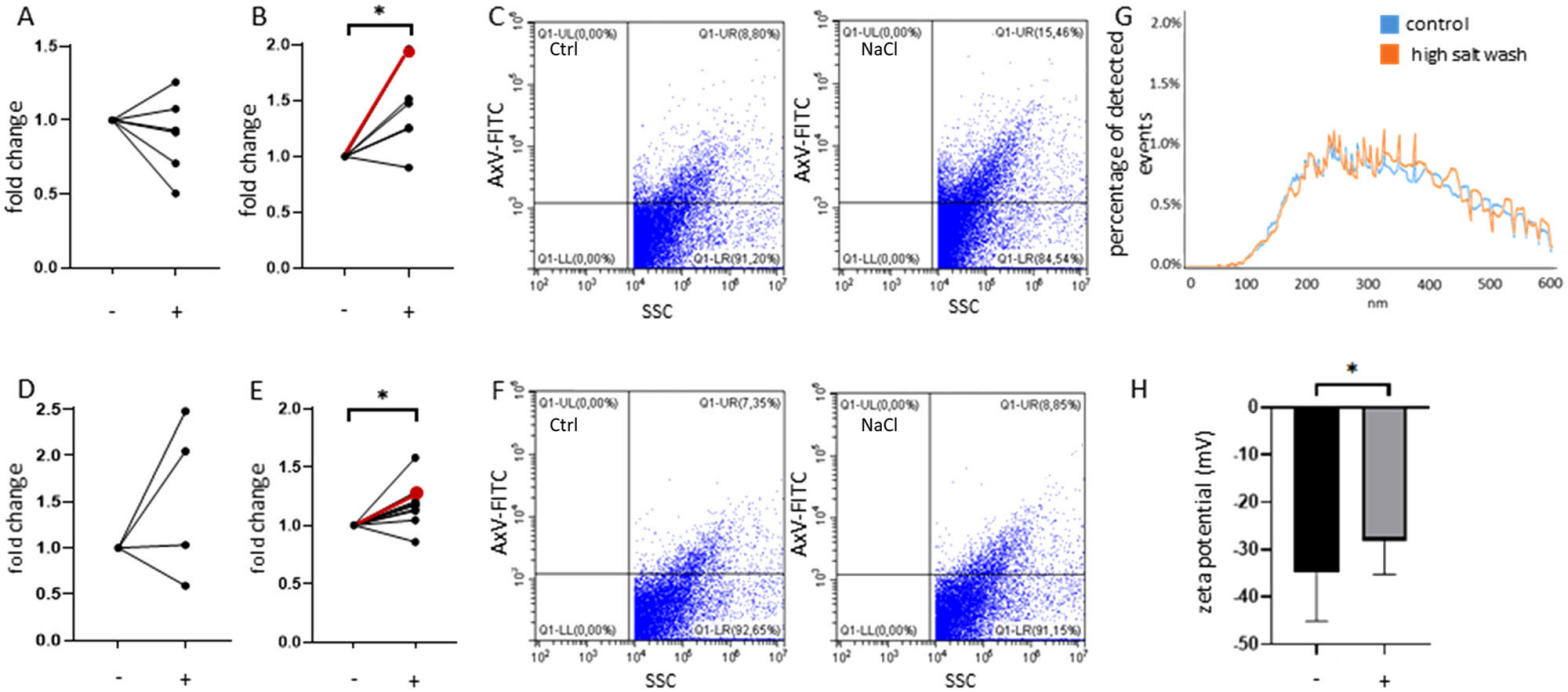
Effect of high‐salt washing on the annexin V positivity of THP1‐derived lEVs.  (A) High‐salt washing (+) did not have an effect on the detection of annexin V‐based affinity capture of THP1 lEVs secreted to serum‐free medium as compared to physiological salt washing (‐) (*n* = 5, Wilcoxon test, *p* = 0.4). (B) In contrast, high‐salt washing (+) increased the fold change of annexin V positive, affinity captured THP1 lEVs secreted to 10% FBS containing medium (*n* = 5, Wilcoxon test, *p* = 0.04). (C) Representative dot plots of affinity captured, serum‐containing medium‐derived lEVs with and without high‐salt washing. Events shown in the representative dot plots are indicated in red in Figure 2B. (D) Annexin V‐FITC labelling of serum‐free medium‐derived THP1 lEVs with (+) and without (‐) high salt washing (*n* = 4, Wilcoxon test). (E) Serum‐containing THP1‐derived lEVs show a significant increase in fold change of the median fluorescence intensity upon high‐salt washing (+) (*n* = 10, Wilcoxon test, *p* = 0.01). (F) Representative flow cytometry plots of annexin V‐FITC labelled, serum‐containing medium‐derived THP1 lEVs with and without high‐salt washing. Data shown in the representative dot plots are indicated in red in Figure 2E. (G) Size distribution curves of serum‐containing THP1‐derived lEVs with and without high‐salt washing (*n* = 11). (H) Zeta potential of serum‐containing medium‐derived lEVs was determined with (+) and without (−) high‐salt washing (*n* = 6, paired *t*‐test, *p* = 0.04).

 To further validate our findings obtained with fluorescently labelled blood plasma proteins associating with EVs, we also carried out experiments using THP1 cell‐derived lEVs. As shown in Figure 2A, when THP1 cells were grown in a serum‐free medium, high‐salt washing did not affect the detection of annexin V affinity captured, bead‐bound lEVs. On the other hand, if the EVs were isolated from 10% FBS‐containing medium (in which plasma protein EV corona formation could occur), a significant increase of affinity bead captured annexin V positive EVs could be detected in the case of the high‐salt washed samples (Figure 2B,C). When using differential centrifugation, a very similar effect was seen in the case of lEVs. When EVs were separated from serum‐free conditioned medium, no difference could be detected in the annexin V positivity after high‐salt washing (Figure 2D). In contrast, when the medium contained FBS, the effect of high‐salt washing resulted in a higher annexin V positivity (Figure 2E,F). We did not detect any difference in the size distributions of high‐salt washed, physiological salt solution‐resuspended and physiological salt‐washed lEVs secreted by THP1 cells in serum‐containing medium (Figure 2G). On the contrary, the zeta potential of lEVs increased significantly (that is the surface charge has become less negative) upon high‐salt washing (*p* < 0.05, paired *t*‐test, Figure 2H), indicating a change in the molecular composition of the EV corona. We also assessed the zeta potential changes of EVs released by two lung cancer cell lines (A549, H1975, Figure S1) upon washing with 1.5 M NaCl solution. In addition, we compared the zeta potential of blood plasma EVs upon LiCl and KCl washes (Figure S2). Even though we did not detect significant differences, we observed a trend toward less negative zeta potentials of EVs after high salt washing.

### 3.3 Alteration of the Protein Corona of lEVs and sEVs Separated From Human Blood Plasma

After analysing THP1 cell line‐derived EVs, we separated circulating lEVs from human PFP samples by annexin V based affinity capture and by differential centrifugation. Annexin V is a protein with high affinity for phosphatidylserine (PS) in the presence of calcium ions. Due to PS externalisation, ectosomal surfaces can be labelled with annexin V and immobilisation of annexin V on magnetic beads enables affinity‐based separation of PS‐exposing particles (Wang et al. 2022).

**FIGURE 3:**
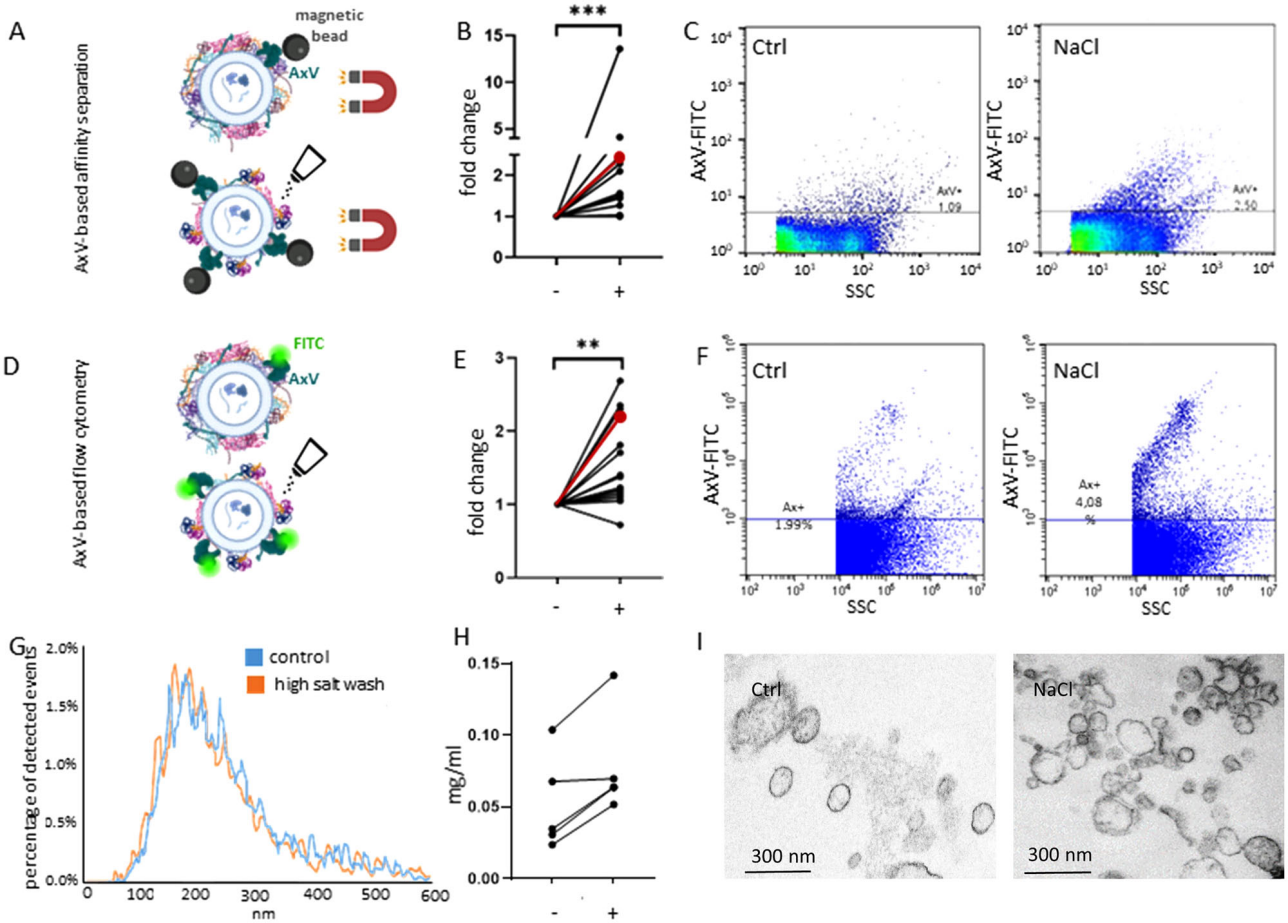
Effect of high‐salt washing on annexin V binding to circulating lEVs. (A) Blood plasma‐derived lEVs were separated by annexin V‐mediated magnetic affinity capture. (B) Fold change of annexin V positive event frequency with (+) and without (−) high‐salt washing (*n* = 13, Wilcoxon test, *p* < 0.001). The values shown in the representative dot plots are indicated in red. (C) Representative flow cytometry plots of annexin V‐FITC positive events with and without high‐salt washing. (D) Blood plasma‐derived lEVs with and without high‐salt washing were labelled by annexin V‐FITC. (E) Fold change of annexin V positive event frequency with (+) and without (−) prior high‐salt washing of lEVs (*n* = 14, Wilcoxon test, *p* < 0.01). The corresponding values shown by the representative dot plots are indicated in red. (F) Representative flow cytometry plots of annexin V‐FITC positive events with and without high‐salt washing of lEVs. (G) Size distributions of lEVs resuspended in NaCl‐HEPES buffer with and without prior high‐salt washing (NTA, *n* = 5). (H) Protein concentrations determined by microBCA in the flowthrough of the annexin V capture column with (+) and without (−) high‐salt washing (*n* = 5, Wilcoxon test, *p* = 0.06). (I) Transmission electron microscopic images of lEVs with and without high‐salt washing.

We isolated lEVs by magnetic‐bead associated annexin V affinity capture, and as shown in Figure 3, high salt washing increased the percentage of annexin V positive events (Wilcoxon test, *p* < 0.001, Figure 3A–C). We found similar results when lEV isolation was carried out by differential centrifugation and the samples were labelled directly with fluorescent annexin V (Wilcoxon test, *p* < 0.01, Figure 3D–F). We also performed NTA and TEM to see whether high‐salt washing altered the particle size distribution or morphology. Figure 3G shows the size distributions of lEVs with and without high salt‐washing and resuspension in physiological salt solution (*n* = 5). Figure 3I demonstrates the ultrastructure of the separated blood plasma‐derived lEVs. After resuspending our EVs in an isosmotic buffer, both control and high‐salt washed lEVs showed characteristic vesicular morphology. After affinity capture of EVs, we measured the protein content of the flow‐through of the columns, and in 4 out 5 samples we found elevated protein concentration in the high‐salt washed samples as compared to the controls (*n* = 5, Wilcoxon test, *p* = 0.06, Figure 3H).

**FIGURE 4:**
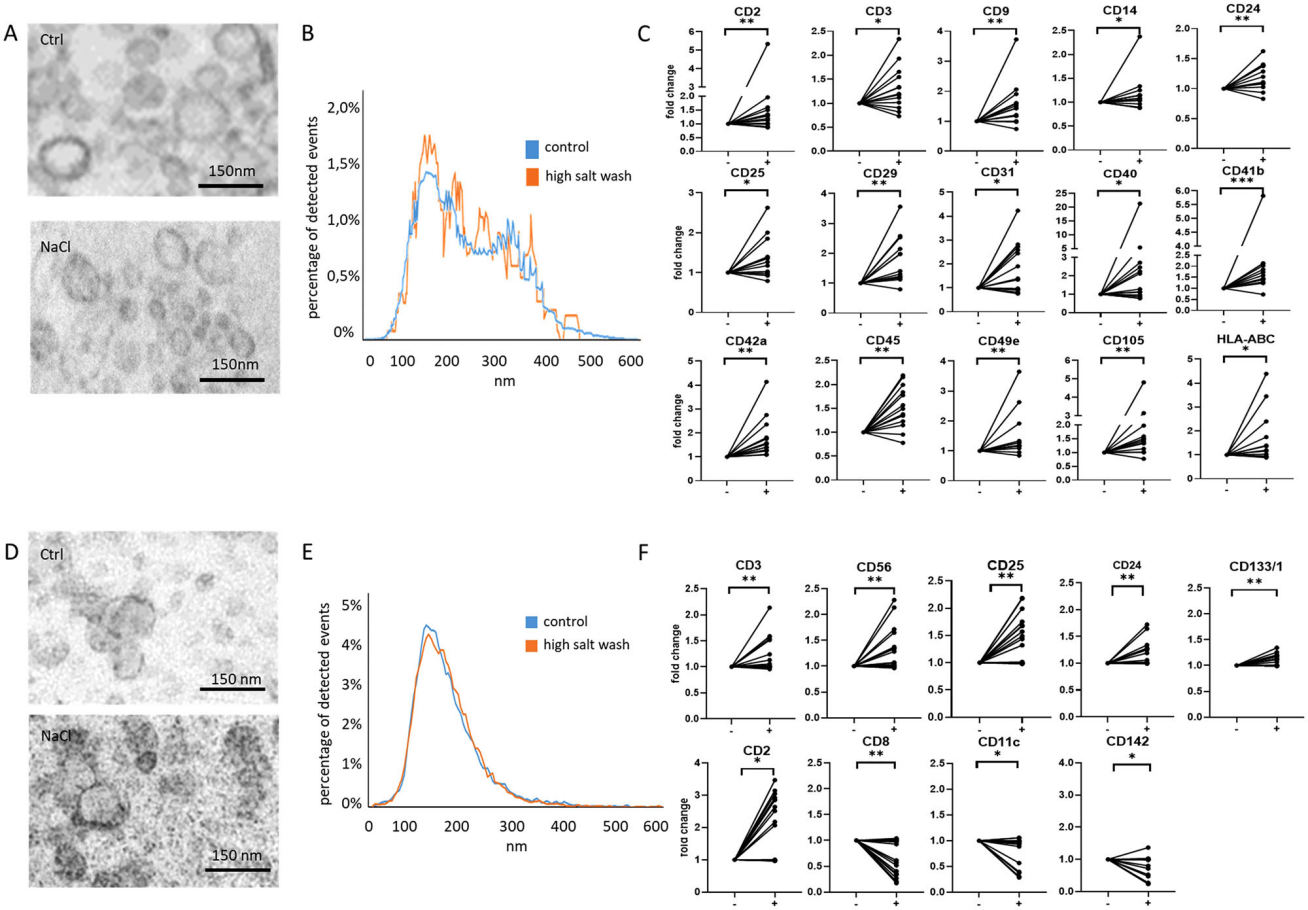
Effect of high‐salt washing on the detection of sEV markers. Healthy human blood plasma‐derived sEVs and THP1‐derived sEVs were separated by differential ultracentrifugation, and were either washed with or without 1.5 M NaCl. Next, 100,000 × *g* pellets were resuspended, and the sEVs were analysed using a MACSPlex kit. (A) Transmission electron microscopic images of plasma sEVs with and without high‐salt washing. (B) Size distribution curves of plasma sEVs with and without high‐salt washing. (C) Fold changes of the median fluorescence intensities of bead‐bound plasma sEV surface molecules showing significant increase upon high‐salt washing (+) as compared to physiological salt solution washing (−) (*n* = 13 plasma donors, *p < 0.05, **p < 0.01, ***p < 0.001, Wilcoxon test). (D) Transmission electron microscopic images of THP‐derived sEVs with and without high‐salt washing. (E) Size distribution curves of THP1‐derived sEVs with and without high‐salt washing. (F) Fold changes of the median fluorescence intensities of bead‐bound THP1 sEV surface molecules showing significant detection upon high‐salt washing (+) as compared to physiological salt solution washing (−) (*n* = 5 isolations, *p < 0.05, **p < 0.01, Wilcoxon test).

After analysing lEVs, our next question was whether high salt washing improved the detection of circulating sEVs. Vesicular structures corresponding to sEVs were clearly visible in the TEM images, regardless of high‐salt washing (Figure 4A). Figure 4B shows no difference between the size distributions of the control and the high‐salt washed plasma sEVs resuspended in physiological salt solution. To assess the accessibility of surface epitopes on sEVs for immunodetection, we used the MACSPlex (Miltenyi Biotec) flow cytometry kit. This kit includes fluorescent beads, each conjugated with antibodies specific to sEV surface molecules. Upon immunocapturing sEVs, an anti‐CD63, CD9 and CD81 antibody cocktail is used for recognition of the sEVs. Out of 37 surface molecules, upon high‐salt washing, we found significantly increased median fluorescence intensities in the case of 15 surface markers (*n* = 13 individual human plasma samples, Figure 4C) (CD3, CD14, CD25, CD31, CD40 and HLA‐ABC *p *< 0.05; CD2, CD9, CD24, CD29, CD45, CD49e and CD105 *p* < 0.01; CD41b and CD42a, *p* < 0.001, Wilcoxon test). In the case of an additional two surface markers, the *p* values ranged between *p* = 0.057 and *p* = 0.07 (CD56 and MCSP). Similarly, no significant difference was detected in the case of the rest of the tested EV surface markers (*n* = 13 individual human plasma samples, Figure S3).

### 3.4 Exposure of Circulating sEVs to EDTA, β‐Mercaptoethanol and Tween‐20

Our attempts to use alternative approaches, for example, washing with EDTA, β‐mercaptoethanol and Tween‐20 or solutions did not affect the detection of human blood plasma‐derived sEV surface molecules using the MACSPlex flow cytometry kit (Figure S4A–C).

### 3.5 Cellular Uptake and Cargo Delivery of sEVs With and Without High‐Salt Washing

To assess if the alteration of the protein corona by high‐salt washing had a functional impact, we analysed the cellular uptake of sEVs, separated from 10% FBS‐containing conditioned medium of HEK293T‐PalmGFP cells. The uptake by THP1 cells did not show a significant difference upon high‐salt washing (*n* = 3, Figure S5). However, in the case of sEVs coated by EV‐depleted PFP of a rheumatoid arthritis patient, we found a significantly reduced uptake upon high‐salt washing (*n* = 3, *p* < 0.01, ANOVA, Figure S5B).

We also investigated the effect of high‐salt washing on the cargo delivery by sEVs. To this end, HEK293T cells edited to express a reporter construct sensitive to Cas9 enzyme, were incubated with FBS‐coated sEVs loaded with the enzyme. High‐salt washing decreased the Cas9 delivery as compared to the control sample (*n* = 3, *p* < 0.01, ANOVA, Figure S5C).

## 4 Discussion

The discovery of the EV corona substantially changed our understanding of contaminants and co‐purified molecular components of EV preparations because the biomolecular corona around EVs may change the biological function or the amount and composition of biomarkers that EVs carry.

Here, we hypothesised that by exposing EVs to potential corona‐stripping conditions, we may modify the protein corona to facilitate EV detection. To validate our hypothesis, first we formed an artificial corona of Cy5‐labelled human blood plasma proteins around membrane‐GFP expressing EVs and confirmed both the formation and the partial removal of some corona proteins by confocal microscopy (Figure 1). Reduction of co‐localisation of the GFP and Cy5 signals suggested loss of corona components upon high salt washing by NaCl, LiCl and KCl solutions. In contrast, EV separation by SEC or washing with Tween‐20, β‐mercaptoethanol and EDTA containing solutions did not have a significant effect. Next, we demonstrated that the flow cytometry‐based annexin V‐FITC detection of both affinity‐captured and differentially centrifuged EVs improved after high‐salt washing. Furthermore, using a MACSPlex flow cytometry kit, we could demonstrate a significantly improved detection of certain blood plasma and THP1‐derived sEV surface markers. Importantly, we found that high‐salt washing did not affect the detection of nascent THP‐1 lEVs produced under serum‐free conditions compared to lEVs released in a serum‐containing medium. This suggests that we did not detect a direct effect of the high‐salt solution on the EV surface marker molecules themselves. In our experiments, NaCl, LiCl and KCl solutions all had considerable effect on the EV corona in contrast to other tested substances. This was similar to our earlier finding that sEV surface‐associated DNA could be removed by high salt washing (Németh et al. 2017). The effect of salt concentration is known to affect self‐diffusion of phospholipids across lipid bilayers (Givens et al. 2019). However, lipid reorientation was not the main reason for our findings (at least not in the case of large EVs), as the effect could be seen only when lEVs were isolated from serum‐containing medium. It is also known that the increased ionic strength of a solution facilitates the disruption of electrostatic molecular interactions (Givens et al. 2019). Ions in high salt solutions may compete with corona proteins for binding sites on the EV surface, thereby reducing the stability of the corona and allowing additional proteins to bind. Competitive adsorption and desorption of plasma proteins have been demonstrated on synthetic nanoparticles (Lee 2021). Another aspect of high ionic strength is its possible impact on the conformation of adsorbed proteins, as it may also cause limited protein unfolding of the corona proteins (Sahin et al. 2010).

Indeed we found an altered zeta potential of EVs upon high salt washing (Figure 2H). We also analysed the amino acid composition of the extracellular domains of EV surface antigens measured by the MACSPlex kit. We hypothesised that disruption of electrostatic interactions by high salt washing may involve proteins with positively charged amino acids in their extracellular domains. Interestingly, we found that in the case of the extracellular domains of proteins with significantly increased detection upon high salt washing, 10 out of 15 had higher arginine (Arg) to total amino acid ratio than the median. This raises the possibility that upon high salt washing, the bonds that the positively charged Arg amino acids formed with corona components, were disrupted (Wake et al. 2024) (Table S2).

On the other hand, high salt washing may induce conformational changes and expose protein domains, which mediate fewer intermolecular interactions. EVs might also reversibly shrink under hyperosmotic conditions and their membrane may physically detach from the protein corona. Thus, during the 12,500 × *g* or 100,000 × *g* centrifugations in high‐salt solution, the shear stress may tear off components of the loosened corona. The significant change in the zeta potential of EVs upon high salt washing could be either the consequence of the loss of proteins from the corona or the aggregation of surface proteins. Therefore, we cannot rule out the possibility that besides stripping off the EV corona, high‐salt washing also induces aggregation of corona proteins thus, exposing bare EV surface areas between the aggregates.

**FIGURE 5:**
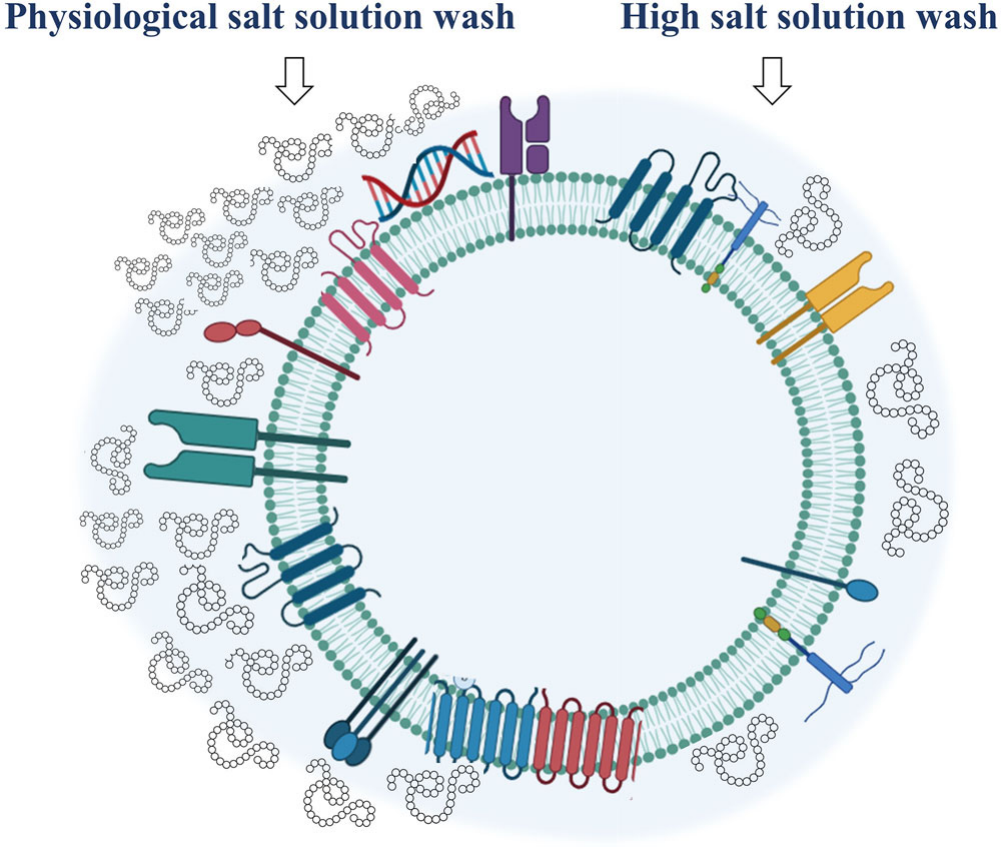
Schematic representation of the effect of high salt washing on the EV corona.

In our current study we found that exposure of EVs to high‐salt washing renders EV surfaces more accessible to annexin V and antibodies. Despite the large number of publications on coronas around artificial nanoparticles (Givens et al. 2019; Böckmann et al. 2003; Brückner et al. 2020), in these studies antibody recognition of the particles themselves was irrelevant. Therefore, our data demonstrating the phenomenon of an improved annexin V and antibody‐based detection of markers upon modification of the corona, is novel and unique to EVs. Our data showing that the EV corona may interfere with antibody and annexin V binding to the EV surface, may also have broader implications. It raises the intriguing possibility that corona components may cover and protect the vesicular surface against immune recognition or enzyme access (Figure 5).

In our uptake and cargo delivery experiments, we found that high salt washing reduced the efficacy of EV uptake and cargo delivery. This implies that corona components may play a role in the internalisation of EVs, potentially altering the uptake pathway and the endosomal escape. This aligns with findings on artificial nanoparticles, which suggest that coronas of different compositions can be recognised differently by cell receptors (Di Santo et al. 2020). Interestingly, in our experiments, high salt washing only reduced the uptake of sEVs significantly when the corona was composed of plasma proteins from an active rheumatoid arthritis patient. This strongly supports the significance of EV corona composition. The results of our uptake and cargo delivery experiments provide evidence for the functional alteration of the EV corona by high salt washing.

High salt washing may have several downstream benefits depending on the purpose of the study. It may reveal EV epitopes and may increase the sensitivity and specificity of EV detection. Therefore, partial removal of the protein corona may enable us to get a better view on EV biology and may facilitate analysis of possible circulating biomarkers improving EV‐based diagnostics. Currently, many different separation protocols and methods are used in the EV field, and these pre‐analytical variables may significantly affect the EV corona composition. Therefore, high salt washing may help standardisation of EV analysis by reducing this variability. This is further supported by the technical reproducibility of the high ionic strength washing approach for EV corona stripping (Figure S6). Finally, by improving the accessibility of the surface of EVs, high salt washing may allow for a better EV surface functionalisation. Taken together, depending on the downstream application, this inexpensive, straightforward and reproducible methodological step can be integrated into protocols for researchers aiming to identify and analyse EV epitopes or EV uptake and delivery.

## 5 Conclusion

In our study, we aimed to investigate if stripping of the EV corona was feasible and whether it could unmask integral EV membrane proteins. Here we demonstrate that by increasing the ionic strength of the EV washing solution, a partial stripping of the EV protein corona can be achieved, thereby increasing the accessibility of surface molecules. This can facilitate the affinity capture isolation, and detection of circulating EVs. Additionally, it enables differential analysis of corona‐coated and partially stripped EVs, along with the potential re‐decoration of stripped EVs by novel functional corona components. Therefore, a high salt washing step may be a valuable addition to studies focusing on EVs and the protein corona.

## Author Contributions

**András I. Försönits**: Conceptualization (equal); investigation (lead); methodology (equal); visualization (lead); writing–original draft (equal). **Eszter Á. Tóth**: Software (equal); visualization (equal)**. Sára Jezsoviczky**: Investigation (equal). **Tünde Bárkai**: Investigation (equal). **Delaram Khamari**: Investigation (equal). **Alicia Galinsoga**: Investigation (equal). **Panna Királyhidi**: Investigation (equal). **Ágnes Kittel**: Investigation (equal). **Júlia Fazakas**: Investigation (equal). **Dorina Lenzinger**: Investigation (equal). **Hargita Hegyesi**: Investigation (equal). **Xabier Osteikoetxea**: Investigation (equal). **Tamás Visnovitz**: Investigation (equal). **Krisztina Pálóczi**: Investigation (equal); methodology (equal). **Szilvia Bősze**: Conceptualization (equal); investigation (equal). Edit I. Buzás: Conceptualization (lead); Investigation (equal).

## Conflicts of Interest

E.I.B. is a member of the Advisory Board of Sphere Gene Therapeutics Inc. (Boston, MA, USA).

## Supporting information




**Supplementary Materials**: jev270124‐sup‐0001‐SuppMat.docx.

## Data Availability

Data available on request from the authors.
