# Supplementary material for: Improved Accessibility of Extracellular Vesicle Surface Molecules Upon Partial Removal of the Protein Corona by High Ionic Strength
Source: J Extracell Vesicles. 2025 Jul 14;14(7):e70124. doi: 10.1002/jev2.70124 (PMC12259388; doi:10.1002/jev2.70124)
Supplement: Supplementary file 1 — Supplementary Materials: jev270124‐sup‐0001‐SuppMat.docx. [file JEV2-14-e70124-s001.docx]

**Supplementary information**

**Improved accessibility of extracellular vesicle surface molecules upon partial removal of the protein corona by high ionic strength**

András I Försönits^1^, Eszter Á Tóth^1,2^, Sára Jezsoviczky^1^, Tünde Bárkai^1^, Delaram Khamari^1^, Alicia Galinsoga 1, Panna Királyhidi^1^, Ágnes Kittel^1^, Júlia Fazakas^1^, Dorina Lenzinger^1^, Hargita Hegyesi^1^, Xabier Osteikoetxea^1,3^, Tamás Visnovitz^1^, Krisztina Pálóczi^1^, Szilvia Bősze^1,4^, Edit I Buzás^1,3,5^

^1^Department of Genetics, Cell- and Immunobiology, Semmelweis University, Budapest, Hungary

^2^The Buda Hospital of the Hospitaller Order of Saint John of God, Budapest, Hungary

^3^HCEMM-SU Extracellular Vesicle Research Group, Budapest, Hungary

^4^HUN-REN-ELTE Research Group of Peptide Chemistry, Budapest, Hungary

^5^HUN-REN-SU Translational Extracellular Vesicle Research Group, Budapest, Hungary

Supplementary Table 1

| **age** | **sex** |
| --- | --- |
| 40 | male |
| 33 | male |
| 31 | male |
| 33 | male |
| 43 | male |
| 33 | male |
| 45 | male |
| 27 | male |
| 33 | female |
| 35 | female |
| 45 | female |
| 36 | female |
| 32 | female |
| 40 | female |
| 43 | female |

**The age and sex of the donors for the blood plasma derived EV analysis.**

Supplementary Table 2

**Amino acids in the extracellular domains of EV surface antigens.**

Amino acid sequences of extracellular vesicle (EV) surface antigens tested by the MACSPlex kit were downloaded from the UniProt database (1). Amino acid composition of the extracellular (EC) domains are shown in the table. If a protein had more than one EC domain, the separate sequences were listed in consecutive rows. The total number of amino acids composing the EC domains are in the first column. Negatively charged (Asp, Glu), positively charged (Arg, Gly, Lys), hydrophobic (Phe, Leu, Ile, Tyr, Trp, Val, Met, Pro) and polar (Ser, Pro, Thr, Cys, Asn, Gln) amino acids were counted and summed numbers are listed in the table. Next, we calculated the ratio of each amino acid type in the given EC domain. In the case of Arginine/total amino acids, values higher than the median value of all proteins listed in the table are indicated in bold. We used the MACSPlex kit to detect the sEV surface markers from blood derived sEVs separated by 100,000 g 70’ ultracentrifugation with or without 1.5 M NaCl washing. For the detection of sEVs on the magnetic beads, we used a cocktail of the common tetraspanin markers CD9, CD63 and CD81 labeled with Allophycocianin (APC). sEV surface markers that showed a significantly improved detection upon 1.5 M NaCl washing are listed above the horizontal line (Wilcoxon test). We could see no differences in the amino acid composition of those markers that showed a significant increase in their fluorescent intensity compared to those markers the detection of which did not improve upon high-salt washing.

Supplementary Figure 1


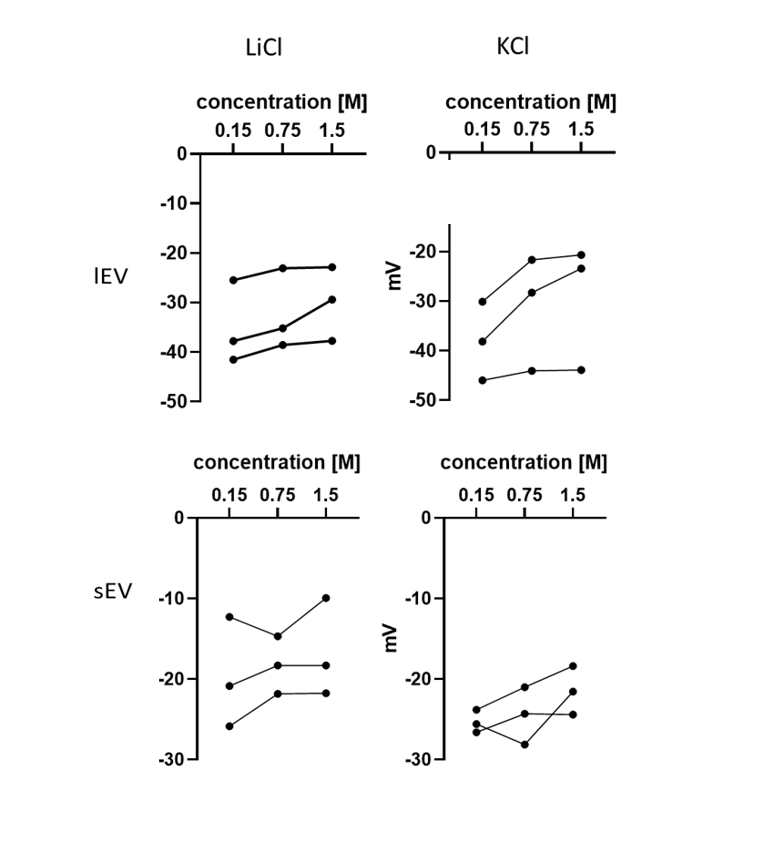


**Zeta potential of blood plasma-derived EVs upon LiCl and KCl washes**

Blood plasma-derived lEVs and sEVs were separated by differential ultracentrifugation from 3 healthy donors. Zeta potential was determined after washing with 0.15M, 0.75M and 1.5M LiCl and KCl. Even though no significant difference was detected (one-way ANOVA). there was a trend toward less negative zeta potentials of EVs after high salt washing

Supplementary Figure 2


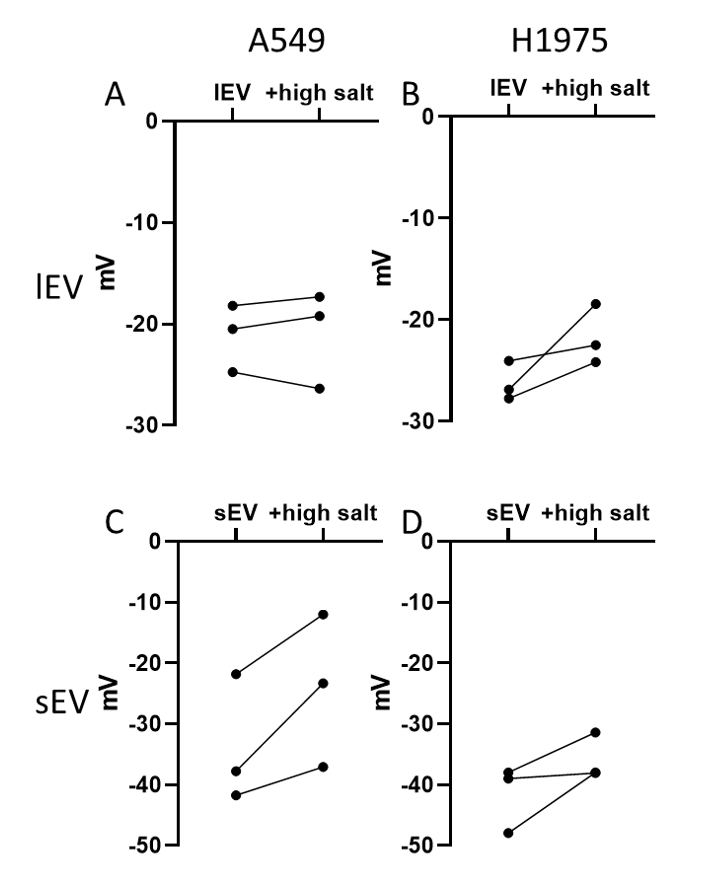


**Zeta potential of lung cancer cell-derived EVs with and without high salt washing**

Large and small EVs were separated from the 10% FBS containing conditioned media of the lung cancer cell lines A459 and H1975 by differential ultracentrifugation: Zeta potential was determined after washing with or without 1.5M NaCl. Student’s paired t-test showed no significant difference, however. there was a trend showing less negative zeta potential of EVs after high ionic strength washing (B-D).


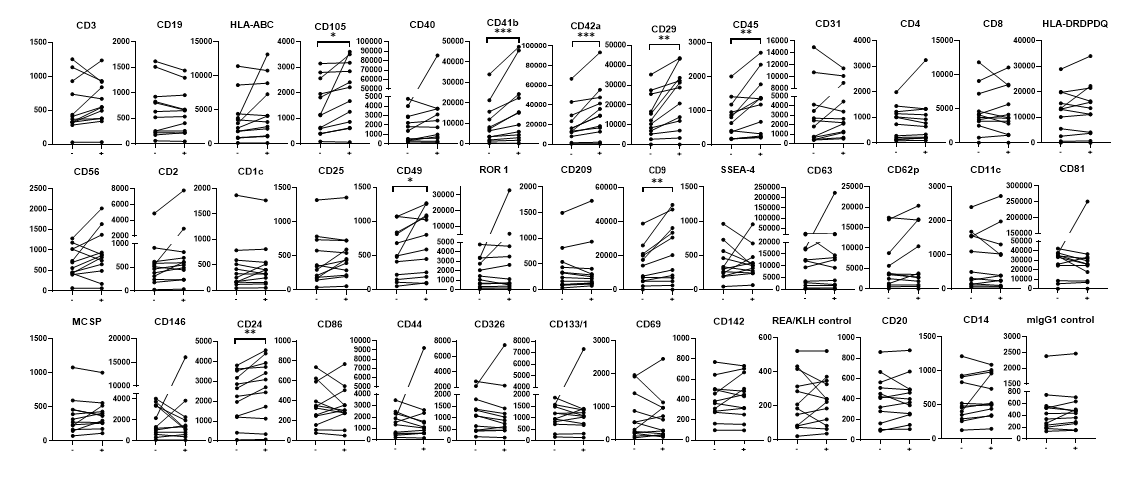
Supplementary Figure 3

**Detection of sEV markers by flow cytometry after high-salt washing.**

We separated blood plasma-derived sEVs by differential ultracentrifugation from 13 healthy donors and used the MACSPlex kit (Miltenyi Biotec) to see if we could alter the recognition of sEV markers upon high-salt washing. sEVs of the same blood sample, washed with 0.9% NaCl and 10 mM HEPES were used as controls (referred to as ‘-‘ in the figure). The median fluorescent intensities were compared to that of the control sEVs. If the measured values showed a normal Gaussian distribution, paired t-test was used. If any of the groups failed the normality test, Wilcoxon matched-pairs signed rank test was applied. Normality was determined by the Shapiro-Wilk test.

Supplementary Figure 4

A - EDTA


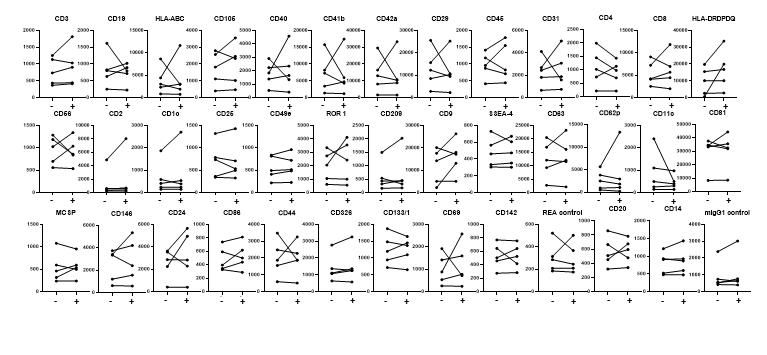


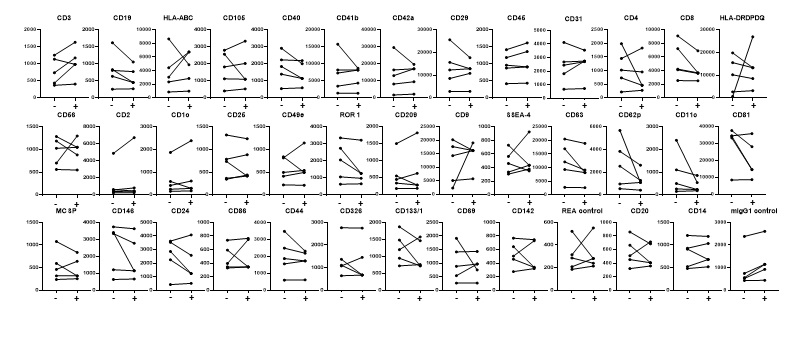

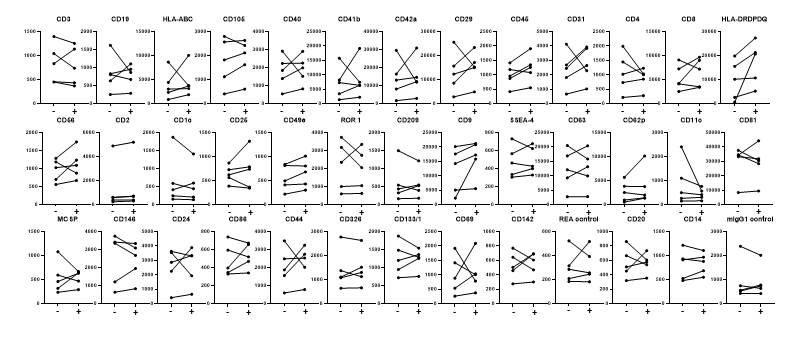


C – Tween-20

B – β-2-mercaptoethanol

**Detection of sEV markers by flow cytometry after washing with different solutions**

We separated blood plasma derived sEVs by differential ultracentrifugation from 5 healthy donors and used the MACSPlex kit (Miltenyi Biotec) to see if we could alter the capture and detection of sEVs by washing with different solutions. We used 5.13 mM ethylenediaminetetraacetic acid (EDTA) (A) or 25 mM β-2-mercaptoethanol (B) or 0.1% Tween-20 (C). sEVs washed with 0.9% NaCl and 10 mM HEPES were used as controls (referred to as ‘-‘ in the figure). The geometric means of the fluorescent intensities were compared to that of the control sEVs (n=5). If the measured values showed a normal Gaussian distribution, paired t-tests were used. If any of the groups failed the normality test, Wilcoxon matched-pairs signed rank test was applied. Normality was determined by the Shapiro-Wilk test.

Supplementary Figure 5


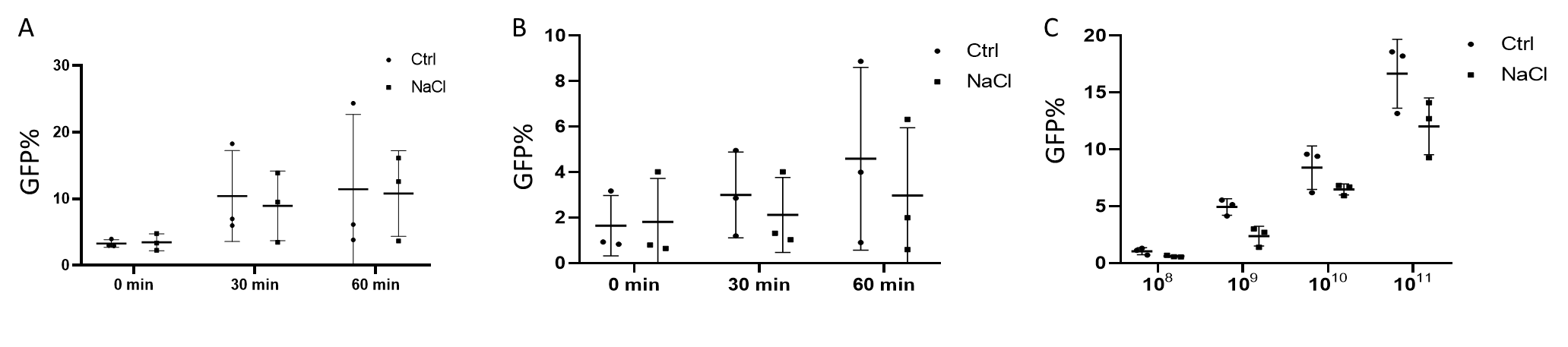


**Effect of high salt washing on the uptake and cargo delivery of sEVs**

The uptake of GFP+ sEVs by THP1 cells was tested. A: sEVs were isolated from the 10% FBS containing conditioned medium of HEK293-PalmGFP cells by differential ultracentrifugation and washed the pellet with physiological salt (Ctrl) or 1.5 M NaCl (NaCl). Next, the sEVs were incubated with THP1 cells and the percentage of GFP positive cells was determined after 0, 30 and 60 minutes by flow cytometry. B: sEVs were isolated from serum-free conditioned medium of HEK293-PalmGFP cells by differential ultracentrifugation. The sEVs were incubated with EV-depleted PFP of a patient with rheumatoid arthritis and washed the corona-coated sEVs with (either with or without) high salt washing. The sEVs were incubated with THP1 cells and the percentage of GFP positive cells was determined after 0, 30 and 60 minutes by flow cytometry. (n=3, p<0.01, ANOVA) C: A HEK293T cell line expressing the Cas9 enzyme was generated along with another one expressing Cas9-sensitive reporter. The sEVs of the Cas9+ cells were isolated from the 10% FBS-containing cell culture medium by differential ultracentrifugation with or without high salt washing. We incubated the reporter expressing HEK293T cells with the sEVs and after 48 hours we assessed the GFP positive cells by flow cytometry. (n=3, p<0.01, ANOVA)

Supplementary Figure 6


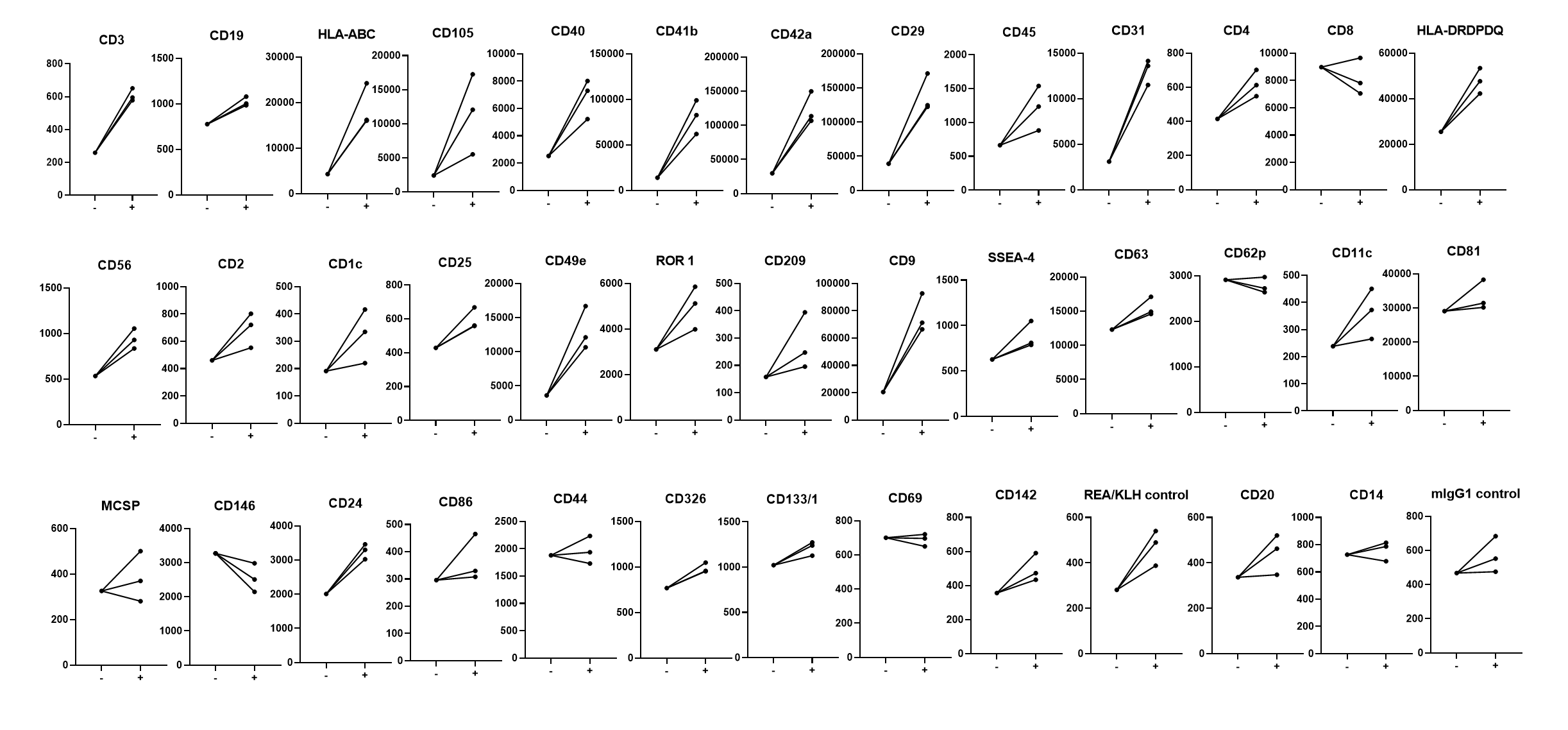


**Technical repeats of the effect of high salt washing of blood plasma sEVs measured by the MACSPlex exosome kit**

We separated blood plasma derived sEVs by differential ultracentrifugation from 13 healthy donors and used the MACSPlex kit (Miltenyi Biotec) to see if we could alter the recognition of sEV markers upon high-salt washing. sEVs of the same blood sample, washed with 0.9% NaCl and 10 mM HEPES were used as controls (referred to as ‘-‘ in the figure). The median fluorescent intensities were compared to that of the control sEVs. If the measured values showed a normal Gaussian distribution, paired t-test was used. If any of the groups failed the normality test, Wilcoxon matched-pairs signed rank test was applied. Normality was determined by the Shapiro-Wilk test.
